# Inhibition of H3K9 methyltransferase G9a ameliorates methylglyoxal-induced peritoneal fibrosis

**DOI:** 10.1371/journal.pone.0173706

**Published:** 2017-03-09

**Authors:** Kazuya Maeda, Shigehiro Doi, Ayumu Nakashima, Takuo Nagai, Taisuke Irifuku, Toshinori Ueno, Takao Masaki

**Affiliations:** Department of Nephrology, Hiroshima University Hospital, Hiroshima, Japan; Hospital Universitario de la Princesa, SPAIN

## Abstract

Activity of H3K9 histone methyltransferase G9a is reportedly induced by transforming growth factor-β1 (TGF-β1) and plays an important role in the progression of cancer and fibrosis. In this study, we investigated whether inhibition of G9a-mediated H3K9 methylation attenuates peritoneal fibrosis in mice and human peritoneal mesothelial cells (HPMCs). Nonadherent cells of peritoneal dialysis (PD) patients were isolated from PD effluent to examine expression of G9a. Peritoneal fibrosis was induced by peritoneal injection of methylglyoxal (MGO) in male C57/B6 mice for 3 weeks. BIX01294, a G9a inhibitor, was administered by subcutaneous injection. Effects of BIX01294 on MGO-induced pathological and functional changes in mice were evaluated by immunohistochemistry and a peritoneal equilibration test. HPMCs were isolated from human omentum, and the inhibitory effect of BIX01294 on TGF-β1-induced fibrotic changes was investigated in the HPMCs by western blotting. G9a was upregulated in nonadherent cells of human PD effluent, the peritoneum of MGO-injected mice, and TGF-β1-stimulated HPMCs. BIX01294 significantly reduced the submesothelial zone thickness and cell density in MGO-injected mice. Immunohistochemical staining revealed that BIX01294 treatment decreased not only mono-methylation of H3K9 (H3K9me1), but also the number of mesenchymal cells, accumulation of collagen, and infiltration of monocytes. In addition to the pathological changes, BIX01294 reduced the level of TGF-β1 in peritoneal fluid and improved peritoneal functions. Furthermore, BIX01294 inhibited TGF-β1-induced fibrotic changes along with suppression of H3K9me1 in HPMCs. Therefore, inhibition of H3K9 methyltransferase G9a suppresses peritoneal fibrosis through a reduction of H3K9me1.

## Introduction

Peritoneal dialysis (PD) is an effective replacement therapy for end-stage kidney disease, and many patients benefit from PD treatment. However, long-term exposure to PD fluid eventually leads to peritoneal fibrosis that is clinically observed as a decrease in water removal [1, 2]. According to previous studies, glucose-driven glucose degradation products (GDPs) participate in this process [3–5]. In fact, among GDPs, the methylglyoxal (MGO) level is reportedly increased in the serum and PD fluid of PD patients, playing a major role in the development of peritoneal fibrosis [6–8]. However, a therapeutic strategy for MGO-induced peritoneal fibrosis has not been established thus far.

Although numerous cytokines have been reported to participate in the progression of peritoneal fibrosis, an increase in transforming growth factor-β1 (TGF-β1) is well known in PD effluents, which plays a pivotal role in this process [9–11]. The pathogenesis of peritoneal fibrosis is characterized by loss of the properties of peritoneal cells, transdifferentiation into myofibroblasts, and production of excessive amounts of extracellular matrix (ECM) [12, 13]. If these processes are classified by transcriptional activity, the loss of cell properties can be classified as decreased transcriptional activity, while fibroblast property acquisition and extracellular matrix protein production can be classified as enhanced transcriptional activity.

Epigenetics are defined as a regulation system of gene expression without changing DNA sequences [14, 15]. A previous study has revealed that changes in gene expression patterns are the true cause of fibrosis, and not changes in DNA sequences [16, 17]. Among epigenetic regulations, methylation of the histone tail is regulated by specific enzymes [18], indicating that TGF-β1-induced histone methyltransferases are therapeutic targets for peritoneal fibrosis. Recently, we have demonstrated that TGF-β1-induced G9a is responsible for renal fibrosis through mono-methylation of lysine 9 in histone H3 (H3K9me1), but not di-methylation (H3K9me2) [19]. G9a-induced H3K9 methylation causes transcriptional silencing [20], raising the possibility that BIX01294, a selective inhibitor of G9a, can suppress the loss of cellular properties and subsequent fibrotic processes through inhibition of H3K9me1.

In this study, we show upregulation of G9a in nonadherent cells isolated from PD effluent, MGO-injected mice, and TGF-β1-induced primary human peritoneal mesothelial cells (HPMCs). We also show that BIX01294 reduces pathological damage and peritoneal dysfunction along with inhibition of H3K9me1 in MGO-injected mice. In HPMCs, BIX01294 attenuates TGF-β1-induced fibrotic changes with a decrease in H3K9me1. Our findings indicate upregulation of G9a in response to TGF-β1 stimulation in not only MGO-injected mice, but also PD patients, and that BIX01294 suppresses peritoneal fibrosis through the reduction of H3K9me1 *in vitro* and *in vivo*.

## Materials and methods

### Animal model

Ten-week-old male C57BL/6 mice weighting of 20–25 g were purchased from Charles River Laboratories Japan (Yokohama, Japan). The animals had free access to laboratory chow and tap water, and were housed in a light- and temperature-controlled room at the Laboratory Animal Center of Hiroshima University (Hiroshima, Japan). The mice were divided into three groups (n = 5 per group) that received (1) intraperitoneal injection of 2.5 mL saline (control mice), (2) intraperitoneal injection of 40 mM MGO (MP Biomedicals LLC, Illkirch, France) + subcutaneous injection of saline (MGO mice), or (3) intraperitoneal injection of 40 mM MGO + subcutaneous injection of 75 μg/day BIX01294 (Sigma-Aldrich, St Louis, MO, USA) (MGO + BIX01294 mice). Peritoneal fibrosis was induced by intraperitoneal injection of 40 mM MGO in 2.5 mL saline [21]. BIX01294 at 75 μg/day in 0.1 mL saline was administrated by subcutaneous injection just before MGO injection. These solutions were administered for 5 consecutive days of the week for 3 weeks.

For the peritoneal equilibrium test (PET), mice were instilled with 4 mL of a PD solution (4.25% Dianeal; Baxter Health Care, Deerfield, IL, USA) before sacrifice by cardiac puncture. After 10 minutes, the peritoneal fluid was removed and blood samples were obtained by cardiac puncture under sedation by sodium pentobarbital anesthesia. The peritoneal permeabilities of glucose and blood urea nitrogen were expressed as the peritoneal absorption of glucose from the dialysate and the dialysate-to-plasma ratio of blood urea nitrogen, respectively. Parietal peritoneum samples were obtained from the contralateral side of injection.

The experimental protocol was approved by the Animal Care and Use Committee of Hiroshima University (permit number: A14-48). All animal experiments were performed in accordance with the National Institutes of Health Guidelines on the Use of Laboratory Animals.

### Histology and immunohistochemistry

Histology and immunohistochemical staining of 4-μm-thick tissue sections were performed as described previously [22]. The following primary antibodies were used: rabbit polyclonal anti-G9a antibody (Abcam, Cambridge, UK), mouse monoclonal anti-α-smooth muscle actin (SMA) antibody (Sigma-Aldrich), rabbit polyclonal anti-collagen I antibody (Abcam), rabbit polyclonal anti-collagen III antibody (Abcam), rat monoclonal anti-mouse CD68 antibody (Serotec, Oxford, UK), rabbit polyclonal anti-TGF-β1 antibody (Santa Cruz Biotechnology, Santa Cruz, CA, USA), and rabbit polyclonal anti-H3K9me1 antibody (Abcam).

The numbers of cells positive for α-SMA, CD68, TGF-β1, and H3K9me1 in the submesothelial compact zone were counted in 10 fields at ×200 magnification. Areas containing collagen I and III were assessed at ×200 magnification in predetermined fields of the submesothelial compact zone captured by a digital camera and analyzed using ImageJ software (version 1.48p; National Institutes of Health, Bethesda, MD, USA) in 10 fields.

### Cell culture of HPMCs

HPMCs were isolated from human omentum as described previously [23]. Harvesting of the omentum was permitted by the Medical Ethics Committee of Hiroshima Graduate School of Biomedical Science (E-84). Written Informed consent was obtained from each patient.

HPMCs were maintained in M199 medium (Life Technologies, New York City, NY, USA) containing 10% fetal bovine serum (FBS) and penicillin/streptomycin. The cells were seeded into six-well plates. At subconfluence, HPMCs were growth arrested for 24 hours in M199 medium containing 0.1% FBS and then treated with 5 ng/mL TGF-β1 (R&D Systems, Minneapolis, MN, USA) for 0–24 hours. Preincubation with 2 μmol/L BIX01294 was carried out for 60 minutes before the 24 hours of TGF-β1 stimulation. We repeated five times of cell culture experiment.

### Western blot analysis and Enzyme-Linked Immunosorbent Assay (ELISA)

Western blot analysis was performed as described previously [24]. Detection of secreted fibronectin was performed as described previously [25]. Each group included five samples. Primary antibodies used in this study were anti-G9a (Cell Signaling Technology, Danvers, MA, USA), anti-α-SMA (Sigma-Aldrich), anti-zonula occludens-1 (ZO-1; Invitrogen, Carlsbad, CA, USA), anti-fibronectin (Sigma-Aldrich), anti-α-tubulin (Sigma-Aldrich), anti-H3K9me1 (Cell Signaling Technology), and anti-H3 (Cell Signaling Technology). The intensity of each band was determined using ImageJ software. Concentrations of TGF-β1 in peritoneal fluid were measured with an ELISA (R&D Systems), following the manufacturer’s instructions.

### Clinical sample collection and ethics statement

To culture nonadherent cells in PD effluent, cells were isolated from spent glucose-based PD fluid (1.5% Dianeal) from three clinically stable PD patients at Hiroshima University Hospital from September 2015 to March 2016. HPMCs were used as the control. This study was approved by the Medical Ethics Committee of Hiroshima Graduate School of Biomedical Science (E-62) and was conducted in accordance with the Declaration of Helsinki. Written informed consent was obtained from each patient.

### Statistical analysis

The results are expressed as the mean ± standard error (SE). Comparisons between two groups were performed using the Student’s t-test, whereas those among three groups were performed by analysis of variance followed by Tukey’s post-hoc test. A value of *P* < 0.05 was considered statistically significant.

## Results

### G9a expression is upregulated in a mouse model of peritoneal fibrosis and in human PD effluent

**Fig 1:**
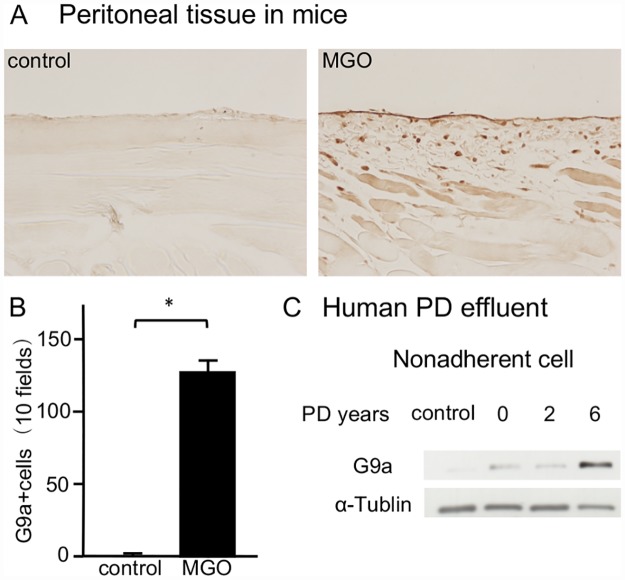
G9a expression is upregulated in MGO-injected mice and human Peritoneal Dialysis (PD) effluents. (A) Immunohistochemical staining shows typical G9a expression in peritoneal tissues of control mice and methylglyoxal (MGO)-injected mice (original magnification, ×200). (B) Graph indicating the number of G9a-positive cells in mice with or without peritoneal injection of MGO (n = 5 for both groups). (C) Representative western blot analysis showing the levels of G9a expression in nonadherent cells of human PD effluents. Data are expressed as the mean ± SE. Statistical analysis was performed by the Student’s t-test. *P < 0.05.

To examine G9a expression in the progression of peritoneal fibrosis, we first performed immunohistochemical staining of G9a in MGO mice. In contrast to few cells expressing G9a in control mice, we found accumulation of G9a-positive cells in the submesothelial zone of MGO mice (Fig 1A and 1B). In nonadherent cells of human PD effluents, we found elevation of G9a expression levels in PD patients compared with HPMCs derived from non-PD patients (Fig 1C).

### BIX01294 suppresses the thickening and accumulation of peritoneal cells induced by MGO

**Fig 2:**
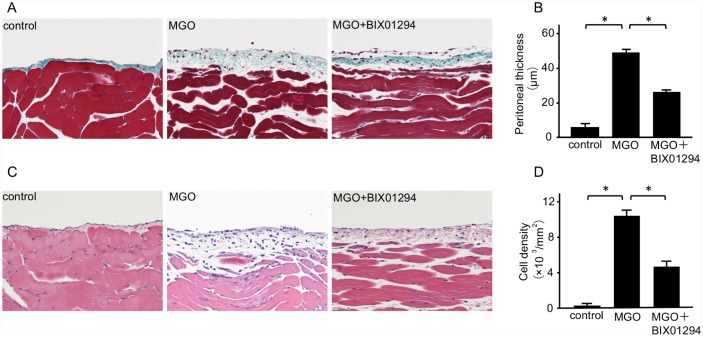
BIX01294 suppresses peritoneal thickening and cell density in MGO-injected mice. (A) Masson’s trichrome staining shows the typical thickness of peritoneal tissue in control mice, MGO-injected mice, and MGO-injected mice treated with BIX01294 (original magnification, ×200). (B) Graph indicates quantification of the peritoneal thickness in the three groups of mice. (C) Hematoxylin-eosin staining shows typical cellularity of peritoneal tissue in control mice, MGO-injected mice, and MGO-injected mice treated with BIX01294 (original magnification, ×200). (D) Graph indicates quantification of cell density in the three groups of mice. Data are expressed as the mean ± SE. Statistical analysis was performed by analysis of variance followed by Tukey’s post-hoc test. *P < 0.05, n = 5 mice per group.

Masson’s trichrome staining was used to analyze the peritoneal thickness, and hematoxylin-eosin staining was used to assess changes in cell density. In control mice, the peritoneal tissues were almost normal without thickening of the submesothelial compact zone. Compared with control mice, the submesothelial compact zone in the peritoneum of MGO mice was significantly thicker and enriched with numerous cells. However, subcutaneous injection of BIX01294 suppressed the thickening of the submesothelial compact zone (Fig 2A and 2B) and the presence of peritoneal cells (Fig 2C and 2D).

### BIX01294 attenuates expression of a mesenchymal marker and ECM proteins in mice with peritoneal fibrosis

**Fig 3:**
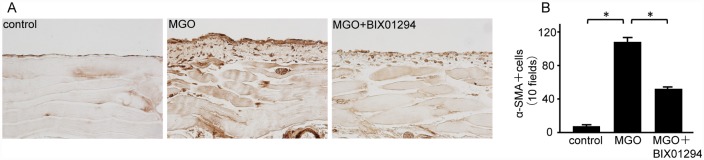
BIX01294 attenuates α-Smooth Muscle Actin (α-SMA) expression in mice with peritoneal fibrosis. (A) Immunohistochemical staining shows typical α-SMA expression in peritoneal tissue of control mice, MGO-injected mice, and MGO-injected mice treated with BIX01294 (original magnification, ×200). (B) Graph indicates the number of α-SMA-positive cells in the three groups of mice. Data are expressed as the mean ± SE. Statistical analysis was performed by analysis of variance followed by Tukey’s post-hoc test. *P < 0.05, n = 5 mice per group.

We examined peritoneal expression of α-SMA as a mesenchymal marker. In MGO mice, numerous α-SMA-positive myofibroblasts were identified in the markedly thickened submesothelial compact zone. BIX01294 administration significantly decreased the number of α-SMA-positive myofibroblasts compared with MGO mice (Fig 3A and 3B).

**Fig 4:**
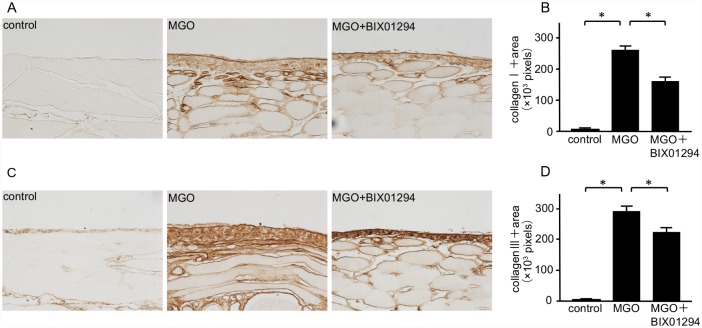
BIX01294 reduces collagen I and III expression in mice with peritoneal fibrosis. (A) Immunohistochemical staining shows typical collagen I expression in peritoneal tissue of control mice, MGO-injected mice, and MGO-injected mice treated with BIX01294 (original magnification, ×200). (B) Graph indicates the number of collagen I-positive pixels in the three groups of mice. (C) Immunohistochemical staining shows typical collagen III expression in the peritoneal tissue of control mice, MGO-injected mice, and MGO-injected mice treated with BIX01294 (original magnification, ×200). (D) Graph indicates the number of collagen III-positive pixels in the three groups of mice. Data are expressed as the mean ± SE. Statistical analysis was performed by analysis of variance followed by Tukey’s post-hoc test. *P < 0.05, n = 5 mice per group.

Collagen I and III are well-known ECM proteins. Although collagen I and III were diffusely expressed in the submesothelial compact zone of MGO mice (Fig 4A and 4C), BIX01294 administration significantly reduced the area of collagen I and III expression (Fig 4B and 4D).

### BIX01294 inhibits infiltration of monocytes/macrophages, TGF-β1 signaling, and G9a-mediated H3K9me1

**Fig 5:**
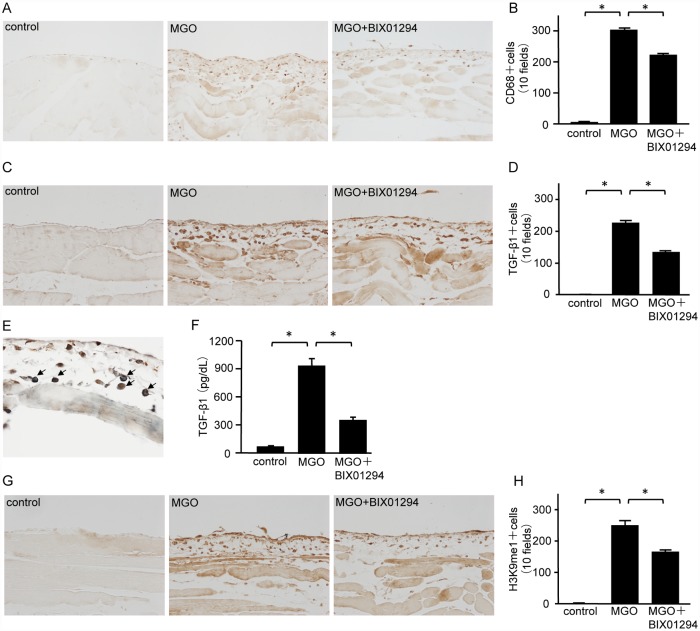
BIX01294 inhibits monocyte/macrophage infiltration, TGF-β1, and H3K9me1 in mice with peritoneal fibrosis. (A) Immunohistochemical staining shows typical CD68 expression in peritoneal tissue of control mice, MGO-injected mice, and MGO-injected mice treated with BIX01294 (original magnification, ×200). (B) Graph indicates the number of CD68-positive cells in the three groups of mice. (C) Immunohistochemical staining shows typical TGF-β1 expression in peritoneal tissue of control mice, MGO-injected mice, and MGO-injected mice treated with BIX01294 (original magnification, ×200). (D) Graph indicates the number of TGF-β1-positive cells in the three groups of mice. (E) Two-color immunohistochemical staining shows that most CD68 cells (brown) are immunoreactive for TGF-β1 (blue-gray) (arrows). (F) TGF-β1 protein levels in mouse PD effluent were quantified by ELISA. (G) Immunohistochemical staining shows typical H3K9me1 levels in peritoneal tissue of control mice, MGO-injected mice, and MGO-injected mice treated with BIX01294 (original magnification, ×200). (H) Graph indicates the number of H3K9me1-positive cells in the three groups of mice. Data are expressed as the mean ± SE. Statistical analysis were performed by analysis of variance followed by Tukey’s post-hoc test. *P < 0.05, n = 5 mice per group.

We examined CD68 expression to investigate monocyte/macrophage infiltration into the peritoneum. The number of CD68-positive cells in the submesothelial compact zone of MGO mice was markedly greater than that in control mice. BIX01294 administration significantly decreased the number of CD68-positive cells compared with MGO mice (Fig 5A and 5B). Similarly, the number of TGF-β1-positive cells in the submesothelial compact zone of MGO mice was markedly greater than that in control mice. Compared with MGO mice, BIX01294 administration decreased the number of TGF-β1-positive cells (Fig 5C and 5D). Most CD68-positive cells showed immunoreactivity for TGF-β1 (Fig 5E, arrows), demonstrating colocalization of CD68 and TGF-β1. The concentrations of TGF-β1 were measured in mouse PD effluent. Protein levels of TGF-β1 in MGO mice were significantly increased in comparison with control mice, but they were dramatically reduced in MGO + BIX01294 mice (Fig 5F). The number of H3K9me1-positive cells in the submesothelial compact zone of MGO mice was markedly larger than that in control mice, and BIX01294 administration significantly decreased the number of H3K9me1-positive cells (Fig 5G and 5H).

### BIX01294 decreases functional impairments of the peritoneal membrane in mice with peritoneal fibrosis

**Fig 6:**
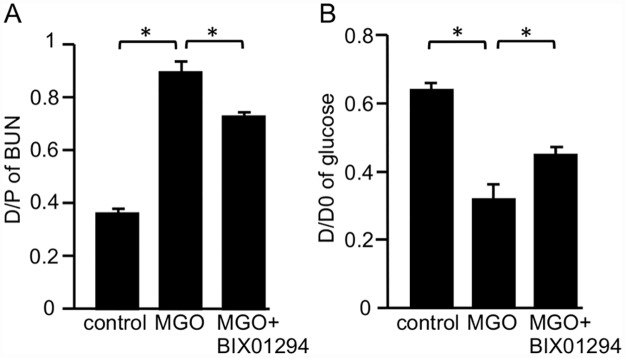
BIX01294 improves functional impairments of the peritoneal membrane in mice with peritoneal fibrosis. Graph shows (A) the dialysate-to-plasma (D/P) ratio of blood urea nitrogen (BUN) and (B) peritoneal absorption of glucose from the dialysate (D/D0) in control mice, MGO-injected mice, and MGO-injected mice treated with BIX01294 during the 10-minute dwell of dialysate (4.25% Dianeal). Data are expressed as the mean ± SE. Statistical analysis were performed by analysis of variance followed by Tukey’s post-hoc test. *P < 0.05, n = 5 mice per group.

A PET was performed to assess functional alterations of the peritoneal membrane. MGO induced a significant increase in permeability for blood urea nitrogen compared with control mice (Fig 6A). In contrast, this change was significantly attenuated in MGO + BIX01294 mice (Fig 6A). In addition, glucose absorption was increased in MGO mice compared with control mice, and significantly improved in MGO + BIX01294 mice (Fig 6B).

### G9a expression is regulated by TGF-β1 in HPMCs and BIX01294 represses TGF-β1-induced fibrotic changes of HPMCs

**Fig 7:**
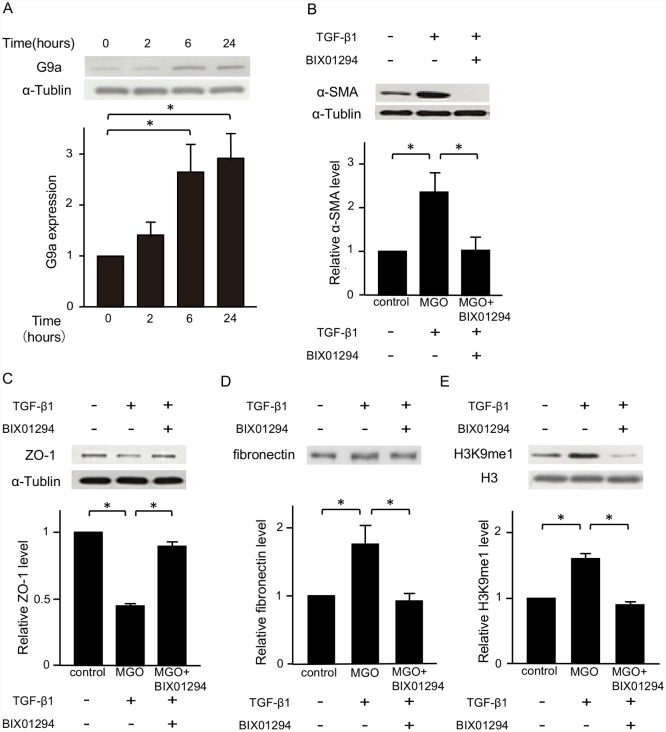
G9a expression is regulated by TGF-β1 in HPMCs, and BIX01294 represses TGF-β1-induced fibrotic changes. (A) Representative western blot analysis showing the levels of G9a protein expression in TGF-β1 (5 ng/mL)-stimulated HPMCs at various time points. Quantification is shown in the lower panel. (B) Representative western blot analysis of the expression of α-SMA (C) zonula occludens-1 (ZO-1), and (D) secreted fibronectin of HPMCs. Quantification is shown in the lower panel. (E) Representative western blot analysis showing the levels of H3K9me1 in TGF-β1-stimulated in HPMCs. Quantification is shown in the lower panel. Data are expressed as the mean ± SE. Statistical analysis was performed by analysis of variance followed by Tukey’s post-hoc test. *P < 0.05, n = 5 samples per group.

TGF-β1 induces fibrotic changes in HPMCs, which are responsible for peritoneal fibrosis and the failure of ultrafiltration. Therefore, we examined the role of TGF-β1 in G9a expression of HPMCs. G9a protein was hardly expressed in control HPMCs, but it was upregulated over time (Fig 7A). Next, to investigate the effects of BIX01294 on fibrotic changes in HPMCs, the cells were stimulated with TGF-β1 with or without BIX01294 for 24 hours. TGF-β1 stimulation increased α-SMA and fibronectin expression, and decreased ZO-1 protein expression. BIX01294 treatment suppressed these TGF-β1-induced fibrotic responses (Fig 7B–7D). The levels of H3K9me1 were increased by TGF-β1 stimulation, and BIX01294 treatment decreased the levels of H3K9me1 (Fig 7E).

## Discussion

In the present study, we found increased expression of G9a in not only a mouse model of MGO-induced peritoneal fibrosis, but also nonadherent cells isolated from PD effluent of patients. We also demonstrated that BIX01294, a selective inhibitor of G9a, suppresses peritoneal fibrosis and peritoneal dysfunctions, which is accompanied by inhibition of H3K9me1. Expression of TGF-β1 increased in the fibrotic area and PD effluent of MGO mice, and its expression was colocalized with infiltrated macrophages. In the *in vitro* experiments, G9a was upregulated by TGF-β1 stimulation and BIX01294 inhibited TGF-β1-induced fibrotic changes in HPMCs along with suppression of H3K9me1. These results suggest that BIX01294 is a candidate therapeutic agent to prevent peritoneal fibrosis through suppression of G9a-mediated H3K9me1.

Among epigenetic changes, histone modifications cause chromatin remodeling, resulting in activation or inactivation of gene expression [26]. Recently, several studies have revealed that histone modification participates in various diseases [27]. In terms of peritoneal fibrosis, previous studies have reported that a histone acetyltransferase inhibitor, C646, suppresses TGF-β1-induced fibrotic changes in HPMCs [28], and that suberoylanilide hydroxamic acid, a histone deacetylase inhibitor, attenuates peritoneal fibrosis in mice via induction of bone morphogenetic protein (BMP)-7 [29]. Notably, our previous report demonstrated that TGF-β1-induced H3K9me1 is responsible for the progression of renal fibrosis [19]. Because histone methylation is regulated by specific enzymes, its regulation is considered to be a more specific therapy for peritoneal fibrosis than regulation of enzymes involved in other histone modifications. Furthermore, G9a is reported to induce di-methylation of H3K9me2 [30], but the level of H3K9me2 was not increased by TGF-β1 stimulation, and BIX01294 treatment did not affect the level of H3K9me2 in HPMCs (data not shown). These findings suggest that pharmacological inhibition of H3K9 methyltransferase G9a ameliorates peritoneal fibrosis through a reduction in H3K9me1.

During the progression of peritoneal fibrosis, TGF-β1-stimulated peritoneal mesothelial cells transdifferentiate into myofibroblasts, contributing to overproduction of ECM proteins. These processes have been experimentally found to be caused by decreased expression of the tight junction protein ZO-1, and increased expression of α-SMA and secretion of fibronectin. The present data show that BIX01294 suppresses the TGF-β1-induced changes in HPMCs along with a reduction of H3K9me1 expression, suggesting that G9a-mediated H3K9me1 regulates ZO-1 expression negatively while regulating expression of α-SMA and fibronectin positively. Moreover, we found an increase of G9a expression in the peritoneum of MGO mice and in human PD effluents. Although we investigated whether TGF-β1 induces G9a expression in the human monocyte/macrophage cell line THP-1, we did not identify any effect of TGF-β1 on G9a expression in inflammatory cells (data not shown). Therefore, detached peritoneal mesothelial cells, but not inflammatory cells, may be the majority of G9a-expressing nonadherent cells.

In this study, we demonstrated that the G9a inhibitor attenuated not only MGO-induced peritoneal fibrosis, but also TGF-β1-stimulated fibrotic changes in HPMCs. Considering that G9a-mediated H3K9me1 reportedly down-regulates transcriptional activity by forming heterochromatin, our data imply a link between transcriptional silencing and fibrosis. In cancer research, several anti-cancer genes have been identified and their silencing leads to the development of tumors. In addition, several anti-fibrotic genes have been reported, such as BMP-7, klotho, and hepatocyte growth factor (HGF) [31–33, 24]. Although we previously found that klotho, but not BMP-7 or HGF, is regulated by G9a-mediated H3K9me1 in human proximal tubular cells (HK-2), we did not find expression of klotho in HPMCs (data not shown). Further studies are needed to elucidate the precise mechanism by which H3K9me1-mediated transcriptional silencing induces fibrosis.

We demonstrated that MGO induces G9a expression through the production of TGF-β1. Although we examined whether MGO directly induces TGF-β1 expression, we did not observe MGO-induced upregulation of TGF-β1 in HPMCs or THP-1 cells (S2 Fig). In addition to TGF-β1, we investigated whether MGO increased the expression of tumor necrosis factor-α and monocyte chemoattractant protein in HPMCs and THP-1 cells. However, we could not identify the mechanism by which MGO induces inflammation (S2 Fig). A possible explanation is that MGO is a precursor of advanced glycation end-products (AGEs) [34]. Increased AGEs may cause inflammation through binding to their receptor [5]. In this study, several double-positive cells for CD68 and TGF-β1 were observed in the submesothelial compact zone. These findings suggest that AGEs induce production of TGF-β1 through activation of inflammatory cells.

In summary, the present data show that TGF-β1 induces G9a, and that G9a-mediated H3K9me1 has major roles in the progression of peritoneal fibrosis. We also show G9a expression in PD patients, although the sample size is small. Furthermore, BIX01294, a G9a inhibitor, attenuates peritoneal fibrosis by suppression of H3K9me1 in MGO-induced mice. In the *in vitro* experiments, BIX01294 treatment also inhibited TGF-β1-induced fibrotic changes by suppression of H3K9me1 in HPMCs. These findings indicate that targeting G9a-mediated H3K9me1 may be an effective strategy to prevent peritoneal fibrosis.

## Supporting information

S1 Fig Mitochondrial activity of HPMCs after treatment with BIX01294. We assessed the cytotoxicity of BIX01294 at 1, 2, and 5 μM in HPMCs by the WST-1 assay that measures mitochondrial activity. The graph shows mitochondrial activity in HPMCs treated with the various doses of BIX01294. Data are expressed as the mean ± SE. Statistical analysis was performed by analysis of variance followed by Tukey’s post-hoc test. *P < 0.05, n = 5 samples per group.
(DOCX)

S2 Fig Expression of cytokines in HPMCs and THP-1 cells under MGO stimulation. Based on previous reports [1, 2], we investigated the effect of MGO on cytokine expression in HPMCs and THP-1 cells. Graphs show MGO-induced MCP-1 expression in HPMCs (a), TNF-α expression in THP-1 cells (b), TGF-β1 expression in THP-1 cells (c) and HPMCs (d), and mRNA expression of TGF-β1 in HPMCs (e). The initial MGO concentration of 100 μM was selected based on the results of a previous report [1]. We tested higher doses of MGO, 100 and 200 μM, in subsequent experiments. Because 200 μM MGO had no effect on the TGF-β1 expression level and another report showing MGO-induced TGF-β1 expression used higher levels of MGO [2], we tested the effects of 300–800 μM MGO on inducing TGF-β1 expression. Lastly, TGF-β1 expression was evaluated by quantitative RT-PCR because it is more sensitive and quantitative than measuring the protein level; this was performed on cells stimulated with 1 mM of MGO. Data are expressed as the mean ± SE. Statistical analysis was performed by analysis of variance followed by Tukey’s post-hoc test. n = 5 samples per group.
(DOCX)
