# Supplementary material for: Inhibition of H3K9 methyltransferase G9a ameliorates methylglyoxal-induced peritoneal fibrosis
Source: PLoS One. 2017 Mar 9;12(3):e0173706. doi: 10.1371/journal.pone.0173706 (PMC5344517; doi:10.1371/journal.pone.0173706)
Supplement: S1 Fig — We assessed the cytotoxicity of BIX01294 at 1, 2, and 5 μM in HPMCs by the WST-1 assay that measures mitochondrial activity. The graph shows mitochondrial activity in HPMCs treated with the various doses of BIX01294. Data are expressed as the mean ± SE. Statistical analysis was performed by analysis of variance followed by Tukey’s post-hoc test. *P < 0.05, n = 5 samples per group. (DOCX) [file pone.0173706.s001.docx]

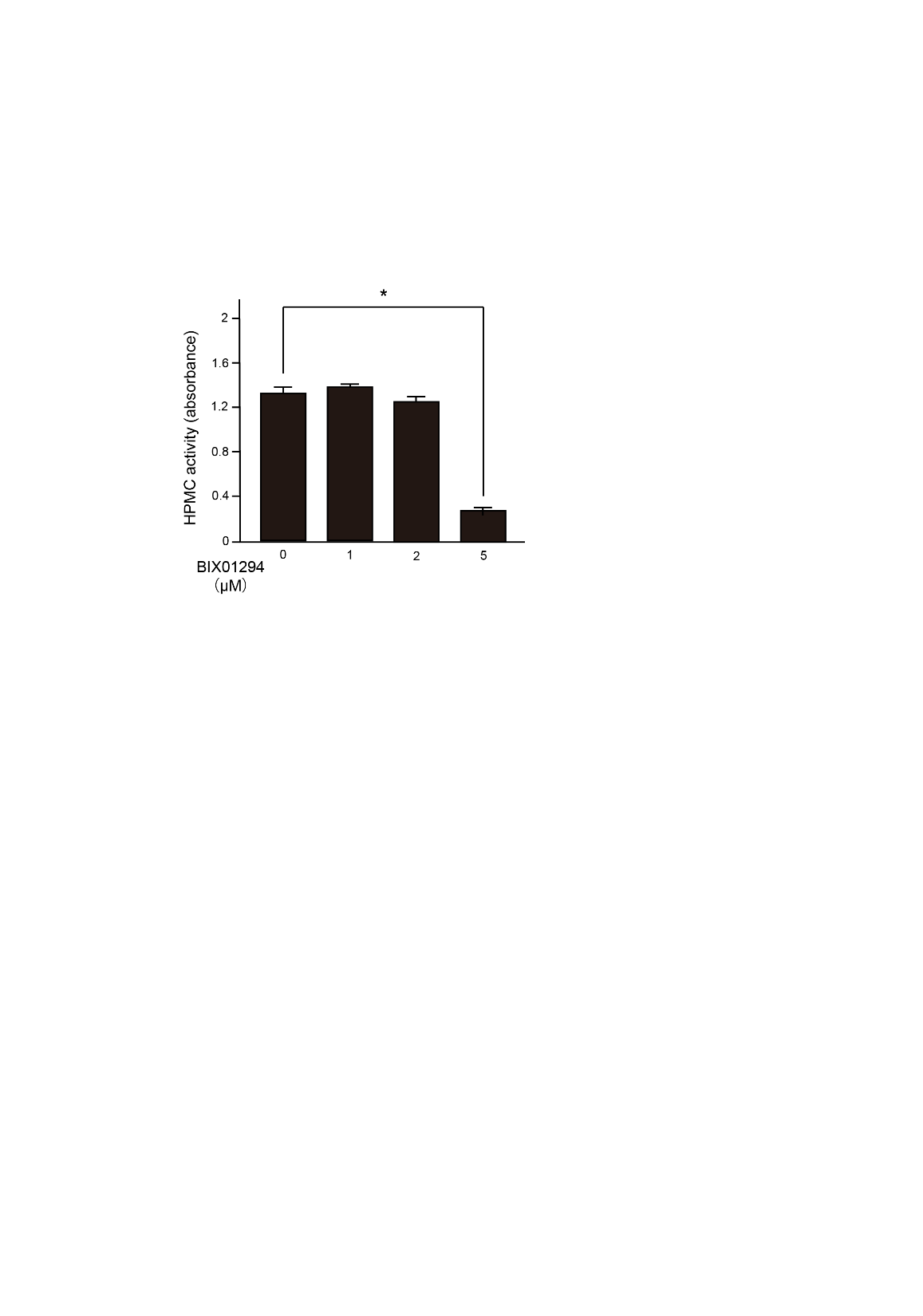


**S1 Fig. Mitochondrial activity of HPMCs after treatment with BIX01294.**

We assessed the cytotoxicity of BIX01294 at 1, 2, and 5 µM in HPMCs by the WST-1 assay that measures mitochondrial activity. The graph shows mitochondrial activity in HPMCs treated with the various doses of BIX01294. Data are expressed as the mean ± SE. Statistical analysis was performed by analysis of variance followed by Tukey’s post-hoc test. **P* < 0.05, n = 5 samples per group.
