# Supplementary material for: Inhibition of H3K9 methyltransferase G9a ameliorates methylglyoxal-induced peritoneal fibrosis
Source: PLoS One. 2017 Mar 9;12(3):e0173706. doi: 10.1371/journal.pone.0173706 (PMC5344517; doi:10.1371/journal.pone.0173706)
Supplement: S2 Fig — Based on previous reports [1, 2], we investigated the effect of MGO on cytokine expression in HPMCs and THP-1 cells. Graphs show MGO-induced MCP-1 expression in HPMCs (a), TNF-α expression in THP-1 cells (b), TGF-β1 expression in THP-1 cells (c) and HPMCs (d), and mRNA expression of TGF-β1 in HPMCs (e). The initial MGO concentration of 100 μM was selected based on the results of a previous report [1]. We tested higher doses of MGO, 100 and 200 μM, in subsequent experiments. Because 200 μM MGO had no effect on the TGF-β1 expression level and another report showing MGO-induced TGF-β1 expression used higher levels of MGO [2], we tested the effects of 300–800 μM MGO on inducing TGF-β1 expression. Lastly, TGF-β1 expression was evaluated by quantitative RT-PCR because it is more sensitive and quantitative than measuring the protein level; this was performed on cells stimulated with 1 mM of MGO. Data are expressed as the mean ± SE. Statistical analysis was performed by analysis of variance followed by Tukey’s post-hoc test. n = 5 samples per group. (DOCX) [file pone.0173706.s002.docx]

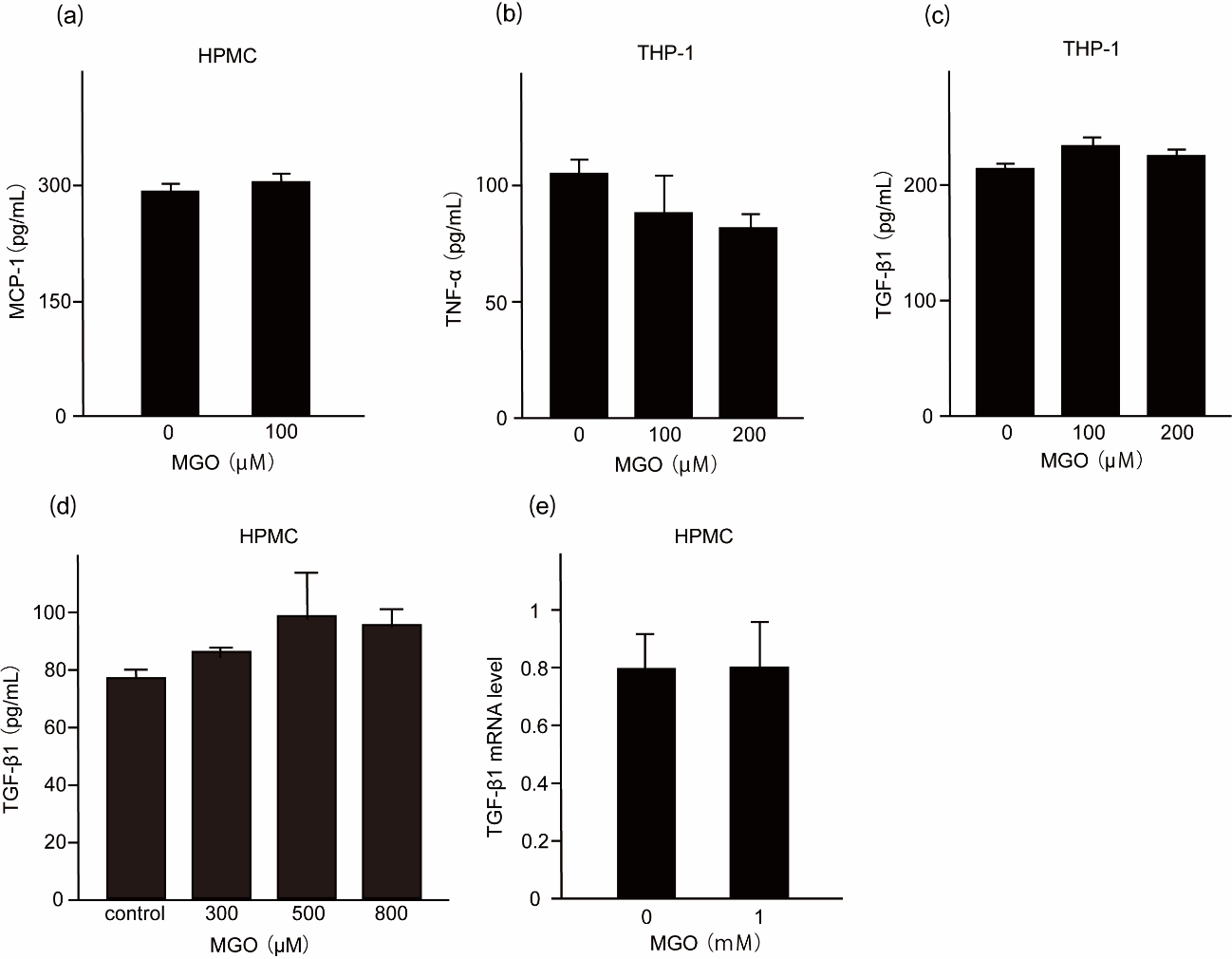


S2 Fig. Expression of cytokines in HPMCs and THP-1 cells under MGO stimulation.

Based on previous reports [1, 2], we investigated the effect of MGO on cytokine expression in HPMCs and THP-1 cells. Graphs show MGO-induced MCP-1 expression in HPMCs (a), TNF-α expression in THP-1 cells (b), TGF-β1 expression in THP-1 cells (c) and HPMCs (d), and mRNA expression of TGF-β1 in HPMCs (e). The initial MGO concentration of 100 µM was selected based on the results of a previous report [1]. We tested higher doses of MGO, 100 and 200 µM, in subsequent experiments. Because 200 µM MGO had no effect on the TGF-β1 expression level and another report showing MGO-induced TGF-β1 expression used higher levels of MGO [2], we tested the effects of 300–800 µM MGO on inducing TGF-β1 expression. Lastly, TGF-β1 expression was evaluated by quantitative RT-PCR because it is more sensitive and quantitative than measuring the protein level; this was performed on cells stimulated with 1 mM of MGO. Data are expressed as the mean ± SE. Statistical analysis was performed by analysis of variance followed by Tukey’s post-hoc test. n = 5 samples per group.

Supporting Information References

1. Hirahara I, Ishibashi Y, Kaname S, Kusano E, Fujita T. Methylglyoxal induces peritoneal thickening by mesenchymal-like mesothelial cells in rats. Nephrol Dial Transplant. 2009;24: 437-447.

2. Hong FY, Bao JF, Hao J, Yu Q, Liu J. Methylglyoxal and advanced glycation end-products promote cytokines expression in peritoneal mesothelial cells via MAPK signaling. Am J Med Sci. 2015;349: 105-109.
